# Comparison of the safety and efficacy of fixed-dose combination of arterolane maleate and piperaquine phosphate with chloroquine in acute, uncomplicated *Plasmodium vivax* malaria: a phase III, multicentric, open-label study

**DOI:** 10.1186/s12936-016-1084-1

**Published:** 2016-01-27

**Authors:** Neena Valecha, Deepali Savargaonkar, Bina Srivastava, B. H. Krishnamoorthy Rao, Santanu K. Tripathi, Nithya Gogtay, Sanjay Kumar Kochar, Nalli Babu Vijaya Kumar, Girish Chandra Rajadhyaksha, Jitendra D. Lakhani, Bhagirath B. Solanki, Rajinder K. Jalali, Sudershan Arora, Arjun Roy, Nilanjan Saha, Sunil S. Iyer, Pradeep Sharma, Anupkumar R. Anvikar

**Affiliations:** National Institute of Malaria Research, Sector 8 Dwarka, New Delhi, 110077 India; Kasturba Medical College, Wenlock District Hospital, Mangalore, India; Department of Pharmacology, Calcutta School of Tropical Medicine, Kolkata, India; Department of Pharmacology, Seth GS Medical College and KEM Hospital, Mumbai, India; Department of Medicine, S.P. Medical College, Bikaner, India; Department of Medicine, Andhra Medical College and King George Hospital, Visakhapatnam, India; Department of Medicine, B.Y.L. Nair Charitable Hospital and T.N. Medical College, Mumbai, India; SBKS Medical College and Dhiraj Hospital, Vadodara, Gujarat India; Department of Medicine, B.J. Medical College, Ahmedabad, Gujarat India; Sun Pharmaceutical Industries Limited (erstwhile Ranbaxy Laboratories Ltd, Gurgaon, India), Gurgaon, India

**Keywords:** Arterolane maleate and piperaquine phosphate, Chloroquine, *Plasmodium vivax*, Primary efficacy analysis, Parasite clearance time, Fever clearance time, Cure rate, Fixed dose combination, Pharmacokinetics

## Abstract

**Background:**

Chloroquine has been the treatment of choice for acute vivax malaria for more than 60 years. Malaria caused by *Plasmodium vivax* has recently shown resistance to chloroquine in some places. This study compared the efficacy and safety of fixed dose combination (FDC) of arterolane maleate and piperaquine phosphate (PQP) with chloroquine in the treatment of uncomplicated vivax malaria.

**Methods:**

Patients aged 13–65 years with confirmed mono-infection of *P. vivax* along with fever or fever in the previous 48 h were included. The 317 eligible patients were randomly assigned to receive FDC of arterolane maleate and PQP (n = 159) or chloroquine (n = 158) for 3 days. Primaquine was given as an anti-relapse measure on day 3 and continued for 14 consecutive days. Primary efficacy analysis included assessment of the proportion of aparasitaemic and afebrile patients at 72 h. Safety endpoints were analysis of adverse events, vital signs, laboratory data, and abnormalities on electrocardiograph. Patients participated in the study for at least 42 days.

**Results:**

In per protocol population, the proportion of aparasitaemic and afebrile patients at 72 h was 100 % (140/140) in the FDC of arterolane maleate and PQP group, and 99.3 % (145/146) in the chloroquine group (Fisher, p > 0.9999). In intent to treat population, the corresponding value was reported to be 96.9 % (154/159) in the FDC of arterolane maleate and PQP group and 98.7 % (156/158) in the chloroquine group (Fisher, p = 0.4479). The median parasite clearance time was 24 h in FDC of arterolane maleate and PQP group and 26 h in chloroquine group (Log-rank, p = 0.2264). Similarly, median fever clearance time was 24 h in both the groups (Log-rank, p = 0.7750). In PP population, day 28 cure rates were 100 % in both the groups (95 % CI (96.52, 100.0 for FDC of arterolane maleate and PQP and 96.73, 100.0 in chloroquine group)). Incidence of adverse events was 82.4 % in the FDC of arterolane maleate and PQP group and 85.4 % in the chloroquine group. Most of the adverse events were mild to moderate in intensity. The commonly reported clinical adverse events in the FDC of arterolane maleate and PQP versus chloroquine group were vomiting (5.0 vs 5.1 %), headache (1.3 vs 3.2 %) and prolonged QT (1.9 vs 3.2 %). No deaths were reported. The pharmacokinetic analysis indicates that arterolane maleate is well absorbed and has a relatively short t_1/2_ of 3.2 h. Piperaquine is also well absorbed after oral administration with a t_1/2_ of about 228.33 h.

**Conclusions:**

The study showed that FDC of arterolane maleate and PQP effectively cured vivax malaria and attained acceptable level of cure up to day 28. Both the groups showed similar safety profile.

*Trial Registration* Clinical Trial Registry India: CTRI/2011/11/002129

**Electronic supplementary material:**

The online version of this article (doi:10.1186/s12936-016-1084-1) contains supplementary material, which is available to authorized users.

## Background

*Plasmodium vivax*, the second most important species causing human malaria, accounts for about 40 % of malaria cases worldwide. It is prevalent in endemic areas in the Middle East, Asia, Oceania, Central and South America. In most areas where *P. vivax* is prevalent, malaria transmission rates are low, and the affected populations, therefore, achieve little immunity to this parasite. Consequently, people of all ages are at risk [1].

In India, chloroquine at a total dose of 25 mg base/kg body weight is the treatment of choice for vivax malaria, since *P. vivax* remains sensitive to chloroquine as shown by the therapeutic efficacy studies [2]. However, sporadic cases of resistance to chloroquine have been reported from India and other countries [3, 4]. In some countries, alternative regimen have been recommended for treatment of vivax malaria [5]. In case there is an emergence of chloroquine resistance in vivax malaria, second-line, alternative anti-malarials should be available.

In some patients, *P. vivax* may cause relapse within a few weeks to a few months after initial infection [6]. For its prevention, primaquine should be given at a dose of 0.25 mg/kg body weight daily for 14 days under supervision [7]. Primaquine is contra-indicated in known G6PD-deficient patients, infants and pregnant women [1, 7–9]. Caution should be exercised before administering primaquine in areas known to have high prevalence of G6PD deficiency [7]. Although chloroquine plus primaquine is the first-line treatment for confirmed vivax malaria in most countries, emergence of chloroquine resistance has been reported from various parts of the world [1, 10, 11]. It is therefore becoming apparent that alternative treatment strategies should be investigated.

It is recommended by the WHO that in areas where artemisinin-based combination therapy (ACT), except artesunate plus sulfadoxine–pyrimethamine, has been adopted as the first-line treatment for falciparum malaria, it may also be used for vivax malaria. Also, in areas with chloroquine-resistant *P. vivax*, ACT (particularly those whose partner medicines have long half-lives) are recommended for the treatment of vivax malaria [1].

Different artemisinin-based combinations (artemether–lumefantrine, dihydroartemisinin–piperaquine, pyronaridine artesunate, artesunate mefloquine) have been evaluated for the treatment of vivax malaria. A Cochrane review showed that dihydroartemisinin–piperaquine (RR 0.32, 95 % CI 0.24–0.43; four trials, 1442 participants) and artesunate plus mefloquine (RR 0.30, 95 % CI 0.21–0.41; four trials, 1003 participants) were more effective than artemether–lumefantrine at reducing the incidence of *P. vivax* over 42 days’ follow-up [12]. A study conducted in Papua, Indonesia demonstrated that both dihydroartemisinin–piperaquine phosphate (DHA–PQP) and artemether–lumefantrine were tolerated and produced rapid clinical response in vivax malaria patients. However, DHA–PQP combination provided better post-treatment prophylaxis than artemether–lumefantrine, reducing *P. vivax* recurrences [13]. The efficacy of PQP has been evaluated in the treatment of vivax malaria. In a study of 280 patients, a total dose of 1.5 g of PQP given over two days was compared with a combination of chloroquine base (1.2 g) and primaquine (30 mg). Both the regimens had equal efficacy in vivax malaria patients [14].

A completely synthetic ozonide anti-malarial based upon the 1,2,4-trioxolane pharmacophore was designed, optimized, developed, and named arterolane. Arterolane exhibits a rapid onset of action, potent activity against all erythrocytic stages of *Plasmodium falciparum*. Arterolane maleate is well absorbed with a t_max_ of 4.5–5.25 h. and has a relatively short t_1/2_ of 2–4 h. The plasma protein binding of arterolane is ~93 % [15]. A 33 % increase in systemic exposure to arterolane has been observed with food. Piperaquine is well absorbed following oral administration with maximum plasma piperaquine concentrations (C_max_) attained at 2.5–4.5 h (T_max_) post dose. The mean half-life ranged from 11 to 18 days  [16]. It is highly bound to plasma proteins (>99 %). Realizing the advantages, arterolane maleate and PQP are combined in a fixed-dose combination (FDC). The tolerability, efficacy and pharmacokinetic profile of piperaquine makes it a promising partner drug for use with short and rapidly acting anti-malarial agents.

In a phase III study conducted with FDC of arterolane maleate and PQP in an adult population, the efficacy of the combination has already been demonstrated against falciparum malaria regardless of geographic region, patient age, gender, or degree of parasitaemia and has been found safe and well tolerated. The drug has already been used for the treatment of patients with falciparum malaria across the wider population in India for more than a year. This phase III study was designed to evaluate efficacy and safety of FDC of arterolane maleate 150 mg and PQP 750 mg in comparison to chloroquine in patients with acute uncomplicated vivax malaria.

## Methods

### Study site and enrolment

This phase III, open-label, multicentric study was conducted from November 2011 to January 2013 at nine clinical trial centres in India. Patients were enrolled at the National Institute of Malaria Research and Bensups Hospital, New Delhi; Calcutta School of Tropical Medicine, Kolkata; BYL Nair Charitable Hospital and TN Medical College Mumbai; Seth GS Medical College and KEM hospital, Mumbai; SP Medical College, Bikaner; SBKS Medical College and Dhiraj Hospital, Vadodara; Kasturba Medical College, Wenlock District Government Hospital, Mangalore; Andhra Medical College and King George Hospital, Visakhapatnam and Civil Hospital, Ahmedabad.

### Inclusion and exclusion criteria

Patients with *P. vivax* mono-infection, aged between 13 and 65 years, weighing ≥40 kg were included into the study after obtaining informed consent/assent, as appropriate. Additional inclusion criteria were parasite density of >250/µl blood, presence of axillary temperature ≥37.5 °C or oral temperature ≥38 °C or fever in the previous 48 h, willingness and ability of patients to comply with the study protocol, and those residing within a reasonable distance of the investigational site. Female patients, of child-bearing potential were included if they were non-lactating and willing to use contraceptive methods during the study period.

Exclusion criteria included mixed *Plasmodium* infection; severe malaria; haemoglobin <8 g/dl; history of haemolytic anaemia or methaemoglobinaemia; pregnant and lactating females; known allergy to artesunate, artemisinin-derived products, PQP, chloroquine, primaquine or any other related drugs; evidence of gastro-intestinal dysfunction that could alter absorption or motility (e.g., diarrhoea defined as >3 episodes of watery stools in the previous 24 h or patients who have had three episodes of vomiting within 24 h prior to screening); use of concomitant medications that could induce haemolysis or haemolytic anaemia or depressants of myeloid element of the bone marrow; any anti-malarial treatment taken during 1 month prior to screening; ongoing prophylaxis with drugs having anti-malarial activity such as cotrimoxazole; participation in any other investigational drug study of at least 3 months prior to screening; any other underlying disease that could compromise the diagnosis and the evaluation of the response to the study medication (including clinical symptoms of immunosuppression, tuberculosis, bacterial infection, cardiac or pulmonary disease); electrocardiogram (ECG) abnormalities with clinical significance or relevance that required urgent management (these abnormalities included QTc interval >450 ms at screening and cardiac conduction disorders, with the exception of right bundle branch block); evidence of significant renal or hepatic impairment (serum creatinine >1.5 ULN aspartate transaminase >2.5 × ULN, alanine transaminase >2.5 × ULN, serum bilirubin >3 mg/dL); splenectomy conducted earlier as confirmed by history or clinical examination; known history of human immunodeficiency virus (HIV) infection or other immunosuppressive disorders; evidence of clinically significant cardiovascular, pulmonary, metabolic, gastrointestinal, neurological, or endocrine diseases, malignancy, or other abnormalities (other than the indication being studied) that could compromise the diagnosis and the evaluation of the response to the study medication; history of epilepsy or convulsions; G6PD-deficient patients; retinal/visual field defects or auditory defects and history of psoriasis and porphyria.

### Ethical considerations

The study was conducted as per the study protocol that was reviewed and approved by the Institutional Ethics Committes/Independent Review Boards of the participating study sites and regulatory authority. This clinical study was conducted in accordance with the Good Clinical Practice, applicable regulatory requirements, and Declaration of Helsinki. Written informed consent was obtained from all patients. In addition, assent was obtained from patients who were <18 years of age wherever it was feasible to the extent of patient’s capabilities. This study is registered with Clinical Trial Registry India, CTRI number CTRI/2011/11/002129.

### Study treatments

Patients were assigned to either of the two study treatments in the ratio of 1:1 according to the randomization schedule. The randomization schedule was stratified by centre in permuted block scheme. Patients were administered three doses of FDC of arterolane maleate 150 mg and PQP 750 mg tablets, and four doses (total of ten tablets of 250 mg each) of chloroquine tablets for three consecutive days. If a patient vomited within 30 min after receiving any dose of investigational products on any of the dosing days, the patient was administered a repeat dose. If the patient vomited again within 30 min after receiving the repeat dose, the patient was withdrawn from the study and given rescue treatment. Re-dosing in case of vomiting was allowed only once during the study.

Following the 3 day treatment period, anti-relapse medication, primaquine tablets 26.3 mg (equivalent to 15 mg base) was issued to patients for consuming orally over 14 consecutive days (one tablet/day). First dose of primaquine phosphate was administered to the patients under supervision on day 3 except in patients who were discharged on day 2 due to personal or social reasons. Compliance to primaquine therapy was confirmed during follow-up days.

### Clinical and laboratory assessments

The study included a pretreatment period (screening day 0), treatment period (days 0 to 2) and post-treatment period (days 3 to 42). The total duration of patient’s participation in the study was at least 42 days following the first dose of study medication. Patients were hospitalized at the clinical trial site for at least three days (days 0, 1 and 2); the hospitalization was extended until patients were aparasitaemic. Patients were discharged on day 3 and they returned for follow-up assessments on days 7, 14, 21, 28, 35, and 42.

At the time of screening, physical examination and clinical assessment was carried out, and vital signs (pulse rate, respiratory rate and Blood Pressure), height, weight and body temperature were measured. Screening assessment also included testing for G6PD deficiency, urine pregnancy test, 12-Lead ECG, parasitological and laboratory investigations.

Physical examination was carried out on days 0, 1, 2 and 3 and at follow-up visits on days 21, 28 and 42. Clinical signs and symptoms, body temperature and vital signs were assessed during the three-day treatment period and at all follow-up visits. The assessments were repeated in case the patient reported with fever. Post-dose ECG measurements were done on day 2 (between two and four hours) after the last dose of either of the investigational products and if abnormal, ECG was recommended to be repeated on subsequent visit or earlier.

Body temperature was recorded at 6 h intervals (or adjusted to the closest possible 6 h interval to make the schedule consistent with routine care) following the first dose of study medication until temperature normalized and remained normal for 24 h, and at every visit thereafter.

Blood smears were prepared and parasite counts measured at screening, predose and at six-hourly intervals following the first dose of study medication until two consecutive negative smears were obtained, thereafter at day 3 and all follow-up visits.

Laboratory parameters (haematology, biochemistry and urinalysis) were assessed on the day of discharge, days 28, 42 or any unscheduled day when required. Pregnancy test was repeated on days 28 and 42.

Any deviations to the study procedures were recorded as protocol deviations. Adverse events were reported for the time of study medication administration and at all follow-up visits. Adverse events reported during the study were adequately followed up until resolution/stabilization or until determination that no further follow up was deemed necessary. Every effort was made by the study staff to follow up adverse events after day 42.

### Drug concentration measurements

The concentration of arterolane, piperaquine, chloroquine and desethylchloroquine in the plasma samples were estimated by LC–MS/MS methods as per US FDA Guidance for Industry: Bioanalytical Method Validation, May 2001 [17]. Analyses were performed in compliance with GLP regulations.

### Blood sampling for pharmacokinetic analysis

A total of nineteen, 1.0 mL venous blood samples were collected from each enrolled patient who were administered with FDC of arterolane maleate and PQP and twenty, 1.0 mL venous blood samples were collected from patients who were administered with chloroquine. The sample collection time points for each schedule are given below:

Sampling schedule A: (FDC of arterolane maleate and PQP)

- Day 0: pre-dose, 0.75, 1.5, 3, 8 and 16 h post first dose.
- Day 2: pre-dose, 1.5, 3, 4, 5, 6, 12 and 24 h post third dose.
- Day 14, day 21, day 28, day 35 and day 42 follow-up visits.

Sampling schedule B: (chloroquine)

- Day 0: pre-dose, 0.75, 1.5, 3, pre-dose (2nd dose), 10 and 16 h post first dose.
- Day 2: pre-dose, 1.5, 2, 3, 4, 6, 12, and 24 h post fourth dose.
- Day 14, day 21, day 28, day 35 and day 42 follow-up visits.

### Bioanalysis of arterolane and piperaquine

A high performance liquid chromatography mass spectrometric method for the simultaneous estimation of arterolane and piperaquine in human plasma was validated as per USFDA guidelines. The method was validated over a concentration range of 1.15–402.72 ng/mL for arterolane and 1.15–403.18 ng/mL for piperaquine. Sample clean-up was accomplished by protein precipitation method using methanol. The samples were analysed by 4000 Q TRAP MS/MS detector in positive ion mode (using a Chromolith SpeedROD RP-18e (50–4.6 mm). The method was found to be sensitive, selective, reproducible, accurate and precise over the above-mentioned range. Matrix effect was also found to be insignificant and the stability of analytes in matrix established successfully.

### Bioanalysis of chloroquine and desethyl chloroquine

A high performance liquid chromatography mass spectrometric method for the simultaneous estimation of chloroquine and desethyl chloroquine in human plasma as per USFDA guidelines. The method was validated over a concentration range of 2.03–502.57 ng/mL for chloroquine and 2.02–501.73 ng/mL for desethyl chloroquine. Sample processing was accomplished by liquid–liquid extraction using ethyl acetate. The samples were analysed by API 4000 MS/MS and 4000 Q TRAP MS/MS detector in positive ion mode (using a Chromolith Performance RP-18e (100–4.6 mm). The method was found to be sensitive, selective, reproducible, accurate and precise over the above-mentioned range. Matrix effect was also found to be insignificant and the stability of analytes in matrix established successfully.

### Pharmacokinetic analysis method

Concentration–time data for arterolane, piperaquine, chloroquine and desethylchloroquine were used to estimate pharmacokinetic parameters using non-compartmental analysis (NCA) in WinNonlin^®^ version 5.3 software.

### Outcomes

The primary efficacy outcome was proportion of aparasitaemic and afebrile patients at 72 h. Secondary efficacy outcome included cure rate on day 28, parasite clearance time (PCT) and fever clearance time (FCT). Cure rate is defined as the absence of parasitaemia irrespective of axillary/oral temperature without previously meeting any of the criteria of treatment failures. PCT is the time in hours from the initiation of therapy until the first of two successive negative smears for parasite is obtained. FCT is the time in hours from the initiation of therapy until disappearance of fever for at least 24 h. Safety endpoints included incidence of adverse events or clinically significant changes in clinical and laboratory parameters, ECG, vital signs, or physical examination findings.

### Statistical considerations

Considering an anticipated cure rate of 90 % at day 3 (proportion of aparasitaemic and afebrile patients at 72 h), 80 % power and non-inferiority margin of 10 %, a total of 284 patients were planned to be evaluated in a ratio of 1:1 (142 evaluable patients in FDC of arterolane maleate and PQP group and 142 evaluable patients in chloroquine group). About 316 patients (including dropout rate of 10 %) were planned to be enrolled to have 284 evaluable patients. Data listings and analyses were performed using Statistical Analysis System (SAS^®^) version 9.1.3, SAS Institute, Cary, NC, USA.

Descriptive statistics for continuous and ordinal variables included mean, standard deviation, 95 % confidence interval of the mean as well as median, minimum, maximum values and quartiles. Categorical variables, such as cure rate, were summarized using count and percentage. Time to event was summarized by number of events, median time and quartiles.

Primary efficacy analysis, i.e., proportion of aparasitaemic and afebrile patients at 72 h was performed for all evaluable (i.e., per protocol population (PP)) and all enrolled (i.e., intent to treat population (ITT)] patients. Secondary efficacy endpoint analysis [PCT, FCT and cure rate at day 28) was performed for all enrolled patients. Cure rate at day 28 was also computed on all evaluable patients. The PP population included all patients who completed a full course of study medication with a known efficacy endpoint and who did not violate the protocol in a way that could affect the efficacy analysis, i.e., the use of prohibited concomitant medication, the presence of significant disease or co-morbid illness, or major protocol violation.

Efficacy proportions were estimated along with their 95 % exact binomial confidence interval for each treatment group and compared between the groups using Fisher exact test and two-sided 95 % Wilson’s confidence interval. Median PCT and FCT was estimated using survival analysis method Kaplan–Meier Graph, and between group, comparison was made using Log-rank test. Patients with early withdrawal or those who could not clear parasite or fever at 72 h were censored at day 7 (168 h), i.e., next follow-up visit. These patients were considered failures in ITT analysis.

Safety analysis was conducted on safety population, i.e., all patients who had received at least one dose of study drug. Analysis of adverse events, laboratory data and other safety evaluations were performed using summary statistics. Data of adverse events was reported in terms of date of onset, intensity or severity, relationship and outcome. Intensity or severity of adverse events was defined using CTCAE version 3.0 toxicity criteria which identifies five grades: mild, moderate, severe, life threatening, and death.

## Results

### Patient disposition, demography and other baseline characteristics

**Fig. 1:**
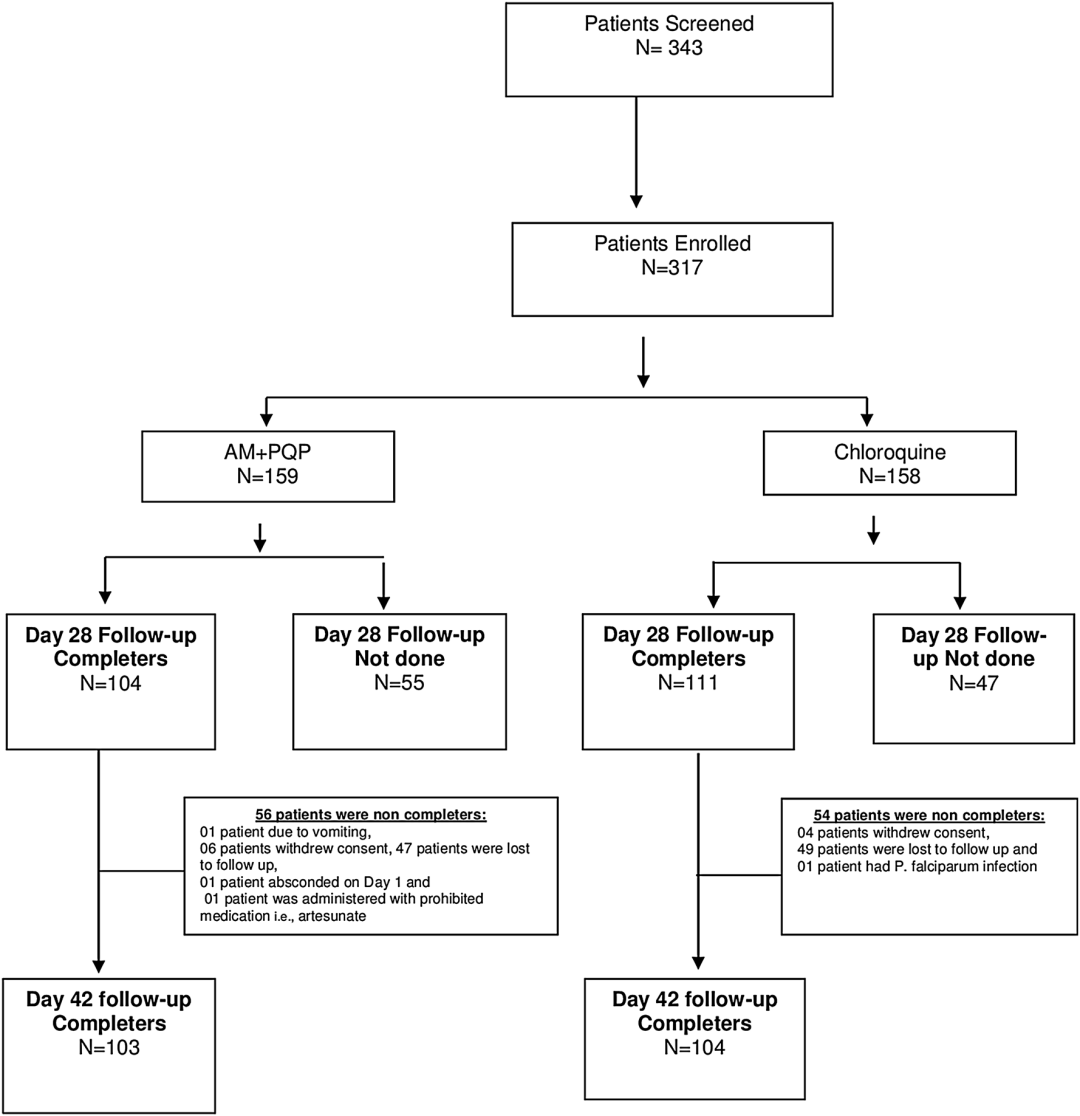
Patients disposition

A total of 317 patients were randomized with 159 patients receiving FDC of arterolane maleate and PQP and 158 patients receiving chloroquine. All patients were included in safety analysis. Two-hundred and seven (207) patients completed the study, i.e., day 42. The patient population was dominated by males 282 (89.0 %). The mean age of the patients was 33.5 ± 12.64 years. Average height and weight of the patients was 163.6 ± 8.24 cm and 58.2 ± 10.25 kg, respectively. Patient demography and baseline characteristics are summarized in Table 1 and patient disposition in Fig. 1.

**Table 1:** Demographic and baseline clinical characteristics

	AM + PQP N = 159	Chloroquine N = 158	Total N = 317
Gender N (%)			
Male	137 (86.2 %)	145 (91.8 %)	282 (89.0 %)
Female	2 2 (13.8 %)	13 (8.2 %)	35 (11.0 %)
Race			
Asian	159 (100.0 %)	158 (100.0 %)	317 (100.0 %)
Age (years)			
Mean ± std	33.2 ± 11.81	33.7 ± 13.45	33.5 ± 12.64
Median	32	30	30
Min, max	13.0, 65.0	15.0, 65.0	13.0, 65.0
Height (cm)			
Mean ± std	163.6 ± 7.83	163.6 ± 8.67	163.6 ± 8.24
Median	164	164	164
Min, max	130.0, 179.0	131.0, 182.0	130.0, 182.0
Weight (kg)			
Mean ± std	57.6 ± 8.76	58.8 ± 11.55	58.2 ± 10.25
Median	57	56.4	57
Min, max	42.0, 81.0	41.2, 128.9	41.2, 128.9
No. of subjects having *P. vivax* asexual parasites (/µL) at screening			
Mean ± std	6458.9 ± 11,439.07	5943.6 ± 10,401.49	6202.9 ± 10,921.57
Median	2704	2880	2750.5
GM	2749.7	2706.5	2728.2
Min, max	250.0, 90,049.0	280.0, 80,000.0	250.0, 90,049.0
Body temperature (degree celsius)			
Mean ± std	38.5 ± 0.62	38.5 ± 0.61	38.5 ± 0.61
Median	38.6	38.6	38.6
Min, max	36.1, 40.2	36.8, 40.4	36.1, 40.4
Fever >(38.0 degree celsius)	122 (76.7 %)	118 (74.7 %)	240 (75.7 %)
No. of subjects having normal temperature (<37.5 degree celsius) at screening	6 (3.8 %)	3 (1.9 %)	9 (2.8 %)
Hepatomegaly	10 (6.3 %)	12 (7.6 %)	22 (6.9 %)
Splenomegaly	10 (6.3 %)	14 (8.9 %)	24 (7.6 %)
Anemia <30 % at screening	12 (7.55 %)	11 (6.96 %)	23 (7.26 %)
Hematocrit at screening			
Mean ± SD	37.14 ± 5.35	37.75 ± 5.30	37.45 ± 5.33
Median	37.4	37.75	37.5
Min, max	19.8, 50	2254.7	19.8, 54.7
G6PD deficiency	0	0	0
*P. vivax* infections in prior 12 months	9 (5.66 %)	8 (5.03 %)	17 (5.36 %)

AM + PQP: FDC of arterolane maleate and PQP

### Efficacy

A total of 286 (90.2 %) patients out of 317 patients were evaluable on day 3 (140 patients in FDC of arterolane maleate and PQP group and 146 patients in chloroquine group). In PP population, all patients (100 %; 140/140) were aparasitaemic and afebrile at 72 h in FDC of arterolane maleate and PQP group compared to 99.3 % (145/146) patients in chloroquine treatment group, (Fisher, p > 0.9999). Both treatments showed efficacy more than 95 % at 72 h, lower 95 % confidence intervals were well above 95 % of success rate in both treatments (Table 2). In ITT population, a total of 154 (96.9 %) out of 159 patients were considered to be aparasitaemic and afebrile at 72 h in FDC of arterolane maleate and PQP group whereas 156 (98.7 %) out of 158 patients were considered to be aparasitaemic and afebrile at 72 h in chloroquine group (Fisher, p = 0.4479). The difference in FDC of arterolane and PQP group was noted in less than 2 % over the chloroquine arm, the lower 95 % confidence limit was well within the prespecified, non-inferiority criteria of −10 % [Table 2, Wilson 95 % CI (−5.99, 1.82)].

**Table 2:** Proportion of patients aparasitemic and afebrile at 72 h (intent to treat population)

Statistical summary	AM + PQP (N = 159)	Chloroquine (N = 158)
Success (n, %)	154 (96.9)	156 (98.7)
95 % CI	(92.81, 98.97)	(95.50, 99.85)
Wilson 95 % CI (AM + PQP—Chloroquine)	−1.9 (−5.99, 1.82)	
Fisher’s exact p value	0.4479	

The secondary variables, day 28 cure rate, PCT and FCT were reported as below: in PP population (n = 215; 104 in FDC of arterolane maleate and PQP group and 111 in chloroquine group), day 28 cure rates were 100 % in both the treatment groups [95 % CI (96.52, 100.0 for FDC of arterolane maleate and PQP and 96.73, 100.0 in chloroquine group)]. Whereas, the results were comparable in ITT population with a total of 109 (68.6 %, 95 % CI 60.72, 75.68) patients out of 159 were considered to be cured on day 28 after administration of FDC of arterolane maleate and PQP and 115 (72.8 %, 95 % CI 65.14, 79.55) patients out of 158 were considered to be cured on day 28 after receiving chloroquine (Table 3). Three patients had reappearance of *P. vivax* at day 42 in choloroquine group but none was seen in the FDC group.

**Table 3:** Cure rate at day 28 (intent to treat population)

Statistical summary	AM + PQP (N = 159)	Chloroquine (N = 158)
Success	109 (68.6)	115 (72.8)
95 % CI	(60.72, 75.68)	(65.14, 79.55)
Wilson 95 % CI (AM + PQP—Chloroquine)	−4.2 (−14.1, 5.77)	
Fisher’s exact p Value	0.4596	

Cure rate at day 28 in PP population was 100 % and has been referred in details in the text

**Fig. 2:**
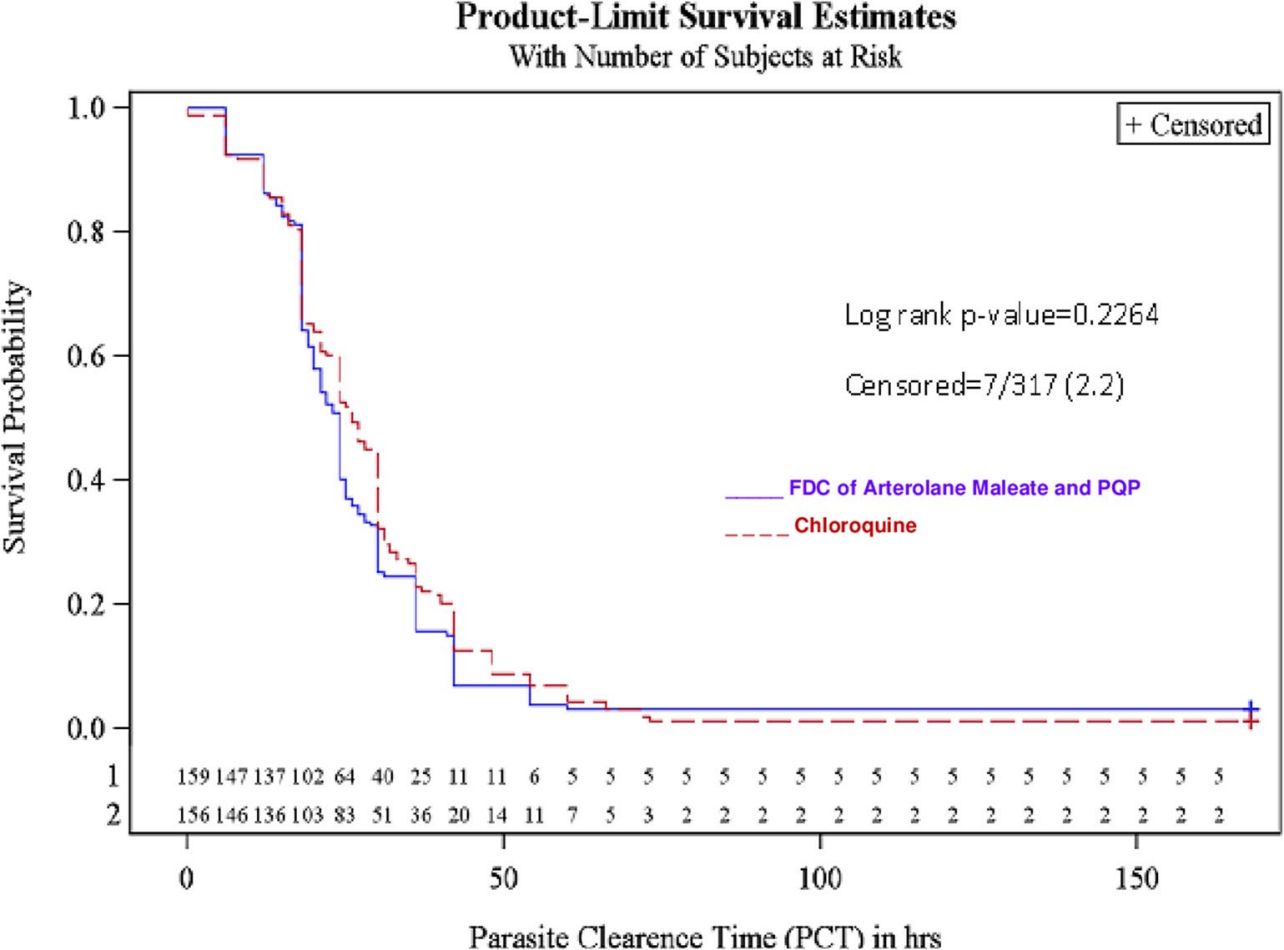
Time to parasite clearance (PCT) Kaplan–Meier method

The median PCT was estimated to be 24 h (18.0–31.0 h) in FDC of arterolane maleate and PQP group whereas PCT of 26 h (18.0–36.0 h) was noted in chloroquine group (Log-rank, p = 0.2264). Both the study treatments demonstrated identical PCT (Table 4 and Fig. 2).

**Table 4:** Parasite clearance time (PCT) (intent to treat population)

Statistical summary	AM + PQP	Chloroquine
Time to parasite clearance (hours)		
Quartile estimate (95 % confidence interval)		
25 %	18.0 (NE, NE)	18.0 (16.0, 18.0)
50 % (median)	24.0 (21.0, 24.0)	26.0 (24.0, 30.0)
75 %	31.0 (30.0, 36.0)	36.0 (30.0, 42.0)
Mean	25.6	28.3
Log-rank test p value	0.2264	

Kaplan–Meier graph, Fig. 2

**Fig. 3:**
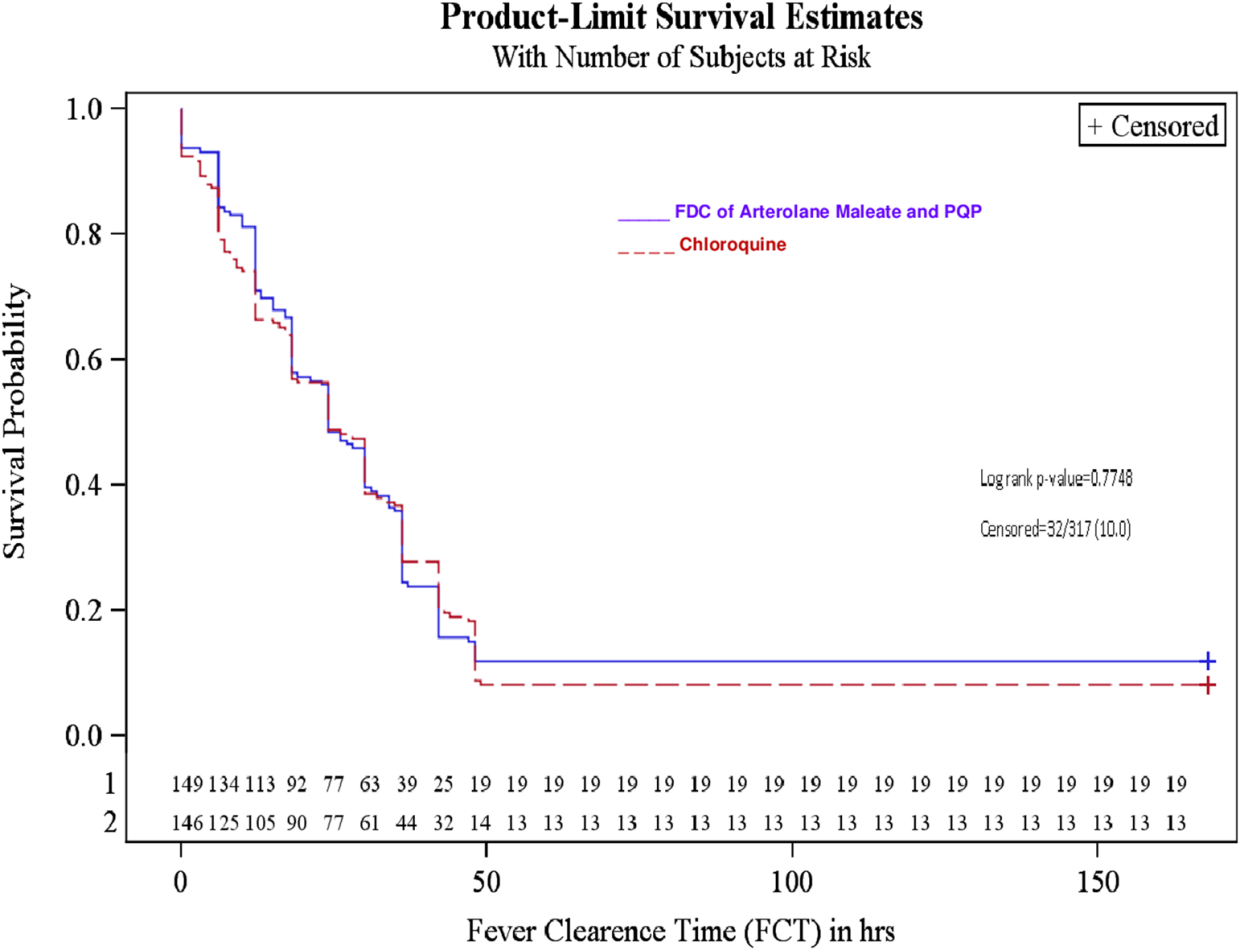
Time to fever clearance (FCT) Kaplan–Meier method

Median FCT in ITT Population was 24 h in both the treatment groups (Log-rank, p = 0.7750, 12–36 h in FDC of arterolane maleate and PQP group and 12–42 h in chloroquine group) (Table 5 and Fig. 3).

**Table 5:** Fever clearance time (FCT) (intent to treat population)

Statistical summary	AM + PQP	Chloroquine
Time to fever clearance (hours)		
Quartile estimate (95 % confidence interval)		
25 %	12.0 (12.0, 18.0)	12.0 (6.0, 12.0)
50 % (median)	24.0 (18.0, 30.0)	24.0 (18.0, 30.0)
75 %	36.0 (36.0, 42.0)	42.0 (36.0, 42.0)
Mean	25.2	25.4
Log-rank test p value	0.7750	

Kaplan–Meier graph, Fig. 3

### Pharmacokinetic results

Pharmacokinetic parameters were evaluated in 89 patients in FDC of arterolane maleate and PQP and 80 patients in chloroquine group.

Arterolane, quantifiable concentration levels were observed up to day 2, which was consistent with a short half-life for this drug. This corroborates with the findings in clinical studies conducted earlier on this combination product. Piperaquine concentrations however, could be quantified until 42 days. This is also expected based on the long terminal half-life of piperaquine. Similarly, chloroquine and desethyl chloroquine, quantifiable concentration levels were observed up to day 42, indicating a long half-life for this drug.

A mean C_max_ of 60.26 ng/mL (ranging from 25.11 to 161.81 ng/ml) and a mean t_1/2_ of 3.20 h (ranging from 0.48 to 8.74 h) was observed for arterolane. Similarly, the mean C_max_ and mean t_1/2_ for piperaquine were 317.82 ng/mL (ranging from 89.13 to 817.14) and 228.33 h (ranging from 10.26 to 757.03 h), respectively.

Chloroquine showed a mean C_max_ of 464.17 ng/mL (ranging from 181.57 to 2467.27 ng/mL) and mean t_1/2_ of 172.47 h (ranging from 12.92 to 757.32 h), while desethylchloroquine exhibited a mean C_max_ of 146.33 ng/mL (ranging from 51.70 to 790.32 ng/mL) and a mean t_1/2_ of 209.06 h (ranging from 17.32 to 525.77 h).

Mean pharmacokinetic parameters along with descriptive statistics for FDC of arterolane maleate and PQP and chloroquine are summarized in Table 6, respectively.

**Table 6:** Mean pharmacokinetic parameters of arterolane, piperaquine, chloroquine and desethylchloroquine

Analyte	Parameters	C_max_ (ng/mL)	AUC_last_ (ng*h/mL)	AUC_48–72_ (ng*h/mL)	t_1/2_ (h)
Arterolane	N	89	89	83	48
	Mean	60.26	1250.09	377.46	3.20
	Minimum	25.11	178.29	15.24	0.48
	Maximum	161.81	3744.98	1100.65	8.74
	% CV	42.23	53.90	62.65	43.06
Piperaquine	N	89	89	84	82
	Mean	317.82	40,424.88	4449.79	228.33
	Minimum	89.13	3548.46	1546.86	10.26
	Maximum	817.14	192,153.39	13,368.70	757.03
	% CV	46.45	72.65	46.03	54.49
Chloroquine	N	80	80	78	76
	Mean	464.17	68,969.77	7474.60	172.47
	Minimum	181.57	10,005.45	3222.40	12.92
	Maximum	2467.27	231,867.11	15,445.88	757.32
	% CV	60.74	48.80	30.15	61.00
Desethylchloroquine	N	78	78	78	78
	Mean	146.33	28,715.98	2625.74	209.06
	Minimum	51.70	2711.74	996.96	17.32
	Maximum	790.32	109,362.90	5931.30	525.77
	% CV	70.33	60.28	41.56	47.29

**Fig. 4:**
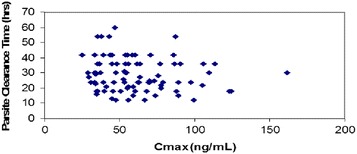
PCT as a function of arterolane C_max_

**Fig. 5:**
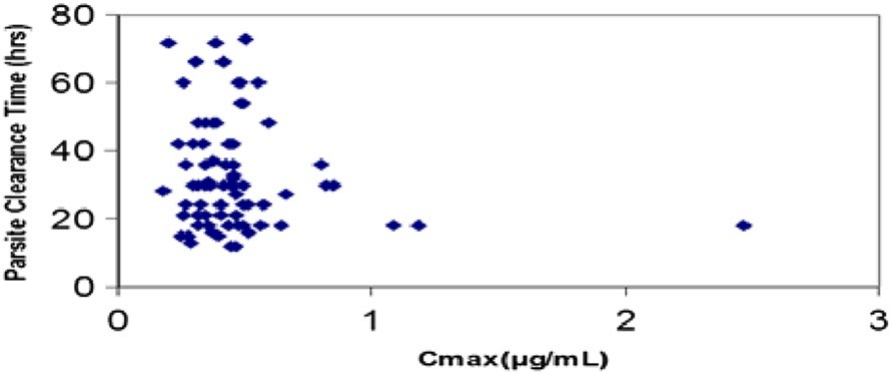
PCT as a function of chloroquine C_max_

**Fig. 6:**
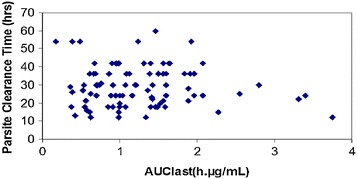
PCT as a function of arterolane exposure (AUC _last_)

**Fig. 7:**
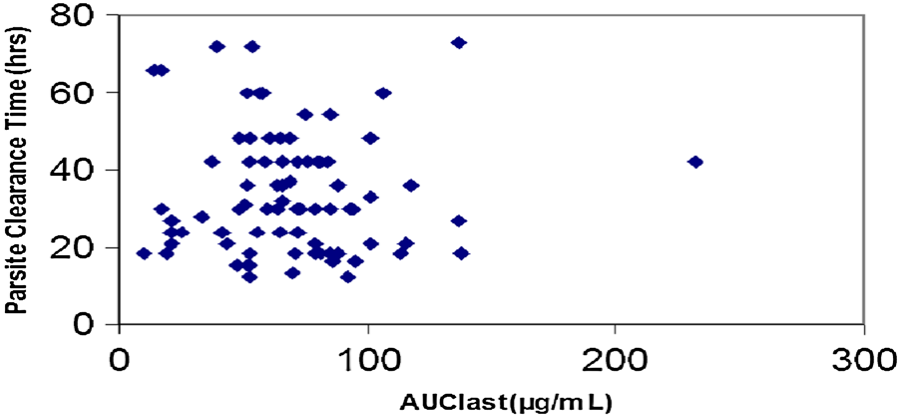
PCT as a function of chloroquine (AUC _last_)

The relationship of PCT versus C_max_ of arterolane and chloroquine is shown in Figs. 4 and 5, respectively. The distribution of PCT appears to be fairly uniform. This indicates that, among the patients evaluated, there is no potential relationship of PCT with C_max_ of arterolane and chloroquine. Exposure (AUC _last_) of arterolane and chloroquine was also plotted against PCT (hours). From the plot, it appears that arterolane exposure levels (AUC _last_) of less than ~2.5 µg.h/ml (Fig. 6) and chloroquine exposure levels (AUC _last_) of less than ~80 µg.h/ml (Fig. 7) are potentially sufficient to result in a PCT ranging from 12 to 60 h and 12 to 73 h, respectively.

### Safety

Incidence of adverse events was 82.4 % (131/159) in FDC of arterolane maleate and PQP and 85.4 % (135/158) in chloroquine group (Additional file 1: Table S1). The majority of adverse events were related to laboratory parameters in both the study groups. Overall, most of the adverse events were mild to moderate in intensity. The commonly reported clinical adverse events in FDC of arterolane maleate and PQP versus chloroquine groups were vomiting (5.0 vs 5.1 %), headache (1.3 vs 3.2 %) and prolonged QT interval (1.9 vs 3.2 %). Four patients in the FDC of arterolane maleate and PQP group had serious adverse events: enteric fever, fever on day 5 (patient was negative for malarial parasite), dehydration and acute glomerulonephritis; all these events were mild to moderate in intensity and resolved without any sequelae. As these patients were hospitalized, the events were considered to be serious. All the events were judged by the investigator as not related to the study medication, except acute glomerulonephritis, which was judged to be related to the study medication. The diagnosis of acute glomerulonephritis was based on clinical judgement and no biopsy was performed. There were no deaths reported during the course of the study.

QTc interval >500 ms was not reported in any of the patients. However, a change in QTc interval of >60 ms over baseline was observed in three patients (1.9 %) from FDC of arterolane maleate and PQP group and five patients (3.2 %) from chloroquine group. The mean QTc interval after Fridericia’s correction was 395.3 ms on day 0 and 401.1 ms on day 2 after treatment with FDC of arterolane maleate and PQP, whereas, the mean QTc interval after Fridericia’s correction was 395.7 ms on day 0 and 406.3 ms on day 2 after treatment with chloroquine (between group comparison, t-test, p = 0.0920). All events of prolongation of QTc interval were judged by the investigators to be mild to moderate in severity. No event was judged to be clinically significant. There were no instances of Torsade de Pointes, sudden death, ventricular tachycardia, ventricular fibrillation and flutter, syncope or seizure in any of the patients which could be identified as a direct outcome of significant prolongation of QTc interval.

Amongst the laboratory parameters, concentration of haemoglobin, platelet count, reticulocyte count, eosinophil count, serum potassium and sodium, ALT and blood glucose values reported on day 0, 3, 28 and 42 were evaluated. Mean concentration/counts were similar after the treatment with arterolane maleate and PQP or chloroquine at these time points.

## Discussion

*Plasmodium vivax* is the dominant species of malaria in many areas outside Africa [18]. Due to emergence of high-grade, multidrug resistance in both *P. falciparum* and *P. vivax* to chloroquine, amodiaquine and sulfadoxine-pyrimethamine, only limited treatment options are available [19].

In this study, 100 % of patients treated with FDC of arterolane maleate and PQP were aparasitaemic and afebrile at day 3 compared to 99.3 % of patients on chloroquine treatment. The efficacy rate of more than 95 % was reported at 72 h in the both treatment groups.

It is recommended by WHO that in areas where ACT, except artesunate plus sulfadoxine-pyrimethamine which is not effective against *P. vivax* in many places, has been adopted as the first-line treatment for falciparum malaria, may also be used for vivax malaria. Also, in areas with chloroquine-resistant *P. vivax*, ACT (particularly the combination having partner medicines with long-half-lives) is recommended for the treatment of vivax malaria [1]. The artemisinin derivatives are the most rapidly acting and potent among anti-malarial medicines [20].

Arterolane maleate is the short-acting component in the FDC of arterolane maleate and PQP, which contributes significantly in achieving early parasite clearance. The time to parasite clearance and fever clearance seen with FDC of arterolane maleate and PQP in this study are on expected lines since the clinical efficacy of the artemisinin derivatives is characterized by an almost immediate onset of activity and rapid reduction of parasitaemia, with complete clearance in most cases within 48 h [21]. Median time to parasite clearance was 24.0 h in FDC of arterolane maleate and PQP group and 26.0 h in chloroquine group. Median FCT was 24.0 h in both the treatment groups.

Day 28 cure rates were 100 % in PP population in both the treatment groups. In ITT population analysis cure rates were reported to be close to 70 % because most of the patients were lost to follow-up, which was considered as failure in the statistical analysis. The possible reason for loss of patients to follow up was migration as the study was conducted in urban settings.

Effectiveness of FDC of arterolane maleate and PQP for *P. vivax* infections would establish a common treatment for both circulating *Plasmodium* species, *P. falciparum* and *P. vivax*. FDC of arterolane maleate and PQP is highly effective against *P. falciparum* based on previously conducted studies [22, 23]. Reports from Thailand and Indonesia detected up to 23 % of *P. falciparum* infections being wrongly diagnosed as *P. vivax*, leading to inappropriate treatment with chloroquine. Treating *P. falciparum* with FDC of arterolane maleate and PQP and primaquine would have the advantage of also treating potential dormant *P. vivax* hypnozoites, which are thought to become activated due to *P. falciparum* infections, thereby explaining the high number of vivax infections after *P. falciparum* treatment [24].

In this study, no deaths were reported and all the four serious adverse events were mild to moderate in intensity and recovered without sequelae. As these patients were hospitalized, the adverse events were considered to be serious in nature. Overall, the incidence of adverse events was similar to incidence reported in patients treated with PQP combinations with DHA in various other studies [12, 13].

The proportion of patients with QTc prolongation of >60 ms from baseline in patients treated with FDC of arterolane maleate and PQP were 1.9 % compared to 3.2 % in chloroquine group. Evaluation of QT interval changes is problematic in malaria because of systematic differences between the acute febrile admission before anti-malarial drugs are given and early convalescence. At presentation, patients are usually anxious, fasting and febrile with increased autonomic tone and a raised heart rate. This is in contrast to the relaxed, fed, supine, afebrile state 3 days later when most anti-malarial treatments finish and anti-malarial concentrations are at the highest. It has been argued that this systematic reduction in sympathetic activity with recovery leads to a consistent increase in the QT interval which has been mistakenly ascribed to anti-malarial drug effects. It has been reported in literature that QTc prolongation of >60 ms is within the bounds of normal daily variation in the QTc (up to 75–100 ms) [25, 26].

Amongst the laboratory parameters, concentration of haemoglobin, platelet count, reticulocyte count, eosinophil count, serum potassium and sodium, ALT and blood glucose were evaluated. Mean concentration/counts reported during laboratory assessments were similar after treatment with FDC of arterolane maleate and PQP or chloroquine. By proportion, the incidence of increased transaminase (ALT and AST) was similar in both the treatment groups. This observation combined with the fact that the FDC of arterolane maleate and PQP is only administered once daily for 3 days suggests that there is a very low risk of liver injury with FDC of arterolane maleate and PQP.

Pharmacokinetic results through non-compartmental analysis indicate that the mean rate and extent of absorption for arterolane maleate and PQP were apparently similar to those observed in patients treated for falciparum malaria. Similarly, the C_max_ and AUC for chloroquine and desethylchloroquine corroborated well with literature-based information [27]. PCT for patients treated with the FDC of arterolane maleate and PQP seems numerically lower than that for the chloroquine. Exposures for chloroquine were higher as compared to piperaquine exposures.

Arterolane (a completely synthetic, ozonide anti-malarial based upon the 1,2,4-trioxolane pharmacophore) in combination with PQP exhibits a rapid onset of action, potent activity against all erythrocytic stages of *P. vivax*. Once-a-day treatment with three doses of FDC of arterolane maleate and PQP efficacy and safety profile similar to chloroquine and no reappearance of vivax malaria noted until day 42 makes this combination a promising candidate, which could lead to better treatment compliance and patient adherence.

## Conclusion

The findings from this study show that FDC of arterolane maleate and PQP effectively cure vivax malaria and attain acceptable level of cure up to day 28. Both the groups have shown similar safety profile.

## Additional file

10.1186/s12936-016-1084-1 Adverse events.

## Abbreviations

ACT: artemisinin-based combination therapy
FDC: fixed-dose combination
AM: arterolane maleate
PQP: piperaquine phosphate
PP: per protocol
ITT: intent to treat
WHO: World Health Organization
RR: risk ratio
DHA: dihydroartemisinin
ECG: electrocardiogram
HIV: human immunodeficiency virus
PCT: parasite clearance time
FCT: fever clearance time
LCMS-MS: liquid chromatography and mass spectrometry’
RP: reversed phase
FDA: food and drug administration
