# Supplementary material for: Comparison of the safety and efficacy of fixed-dose combination of arterolane maleate and piperaquine phosphate with chloroquine in acute, uncomplicated Plasmodium vivax malaria: a phase III, multicentric, open-label study
Source: Malar J. 2016 Jan 27;15:42. doi: 10.1186/s12936-016-1084-1 (PMC4728808; doi:10.1186/s12936-016-1084-1)
Supplement: Supplementary file 1 — 10.1186/s12936-016-1084-1 Adverse events. [file 12936_2016_1084_MOESM1_ESM.doc]

**Table 7: Adverse Events**

| **Preferred Term** | **AM + PQP (N=159)** | **Chloroquine**  **(N=158)** |
| --- | --- | --- |
| **No. of subjects with at least one AE** | 131 (82.4%) | 135 (85.4%) |
| **Total no. of AEs** | 383 | 438 |
| Lymphocytosis | 1 (0.6%) | 0(0.0%) |
| Sinus bradycardia | 1 (0.6%) | 0(0.0%) |
| Abdominal pain upper | 1 (0.6%) | 0(0.0%) |
| Diarrhoea | 0 (0.0%) | 1(0.6%) |
| Gastritis | 0 (0.0%) | 2(1.3%) |
| Nausea | 2 (1.3%) | 3(1.9%) |
| Vomiting | 8 (5.0%) | 8(5.1%) |
| Asthenia | 1 (0.6%) | 0(0.0%) |
| Chills | 0 (0.0%) | 2(1.3%) |
| Fatigue | 2 (1.3%) | 0(0.0%) |
| Pyrexia (p=0.0137) | 6 (3.8%) | 0(0.0%) |
| Swelling | 0 (0.0%) | 1(0.6%) |
| Filariasis | 1 (0.6%) | 0(0.0%) |
| Herpes simplex | 0 (0.0%) | 1(0.6%) |
| Herpes zoster | 0 (0.0%) | 1(0.6%) |
| Oral herpes | 1 (0.6%) | 2(1.3%) |
| Otitis externa | 0 (0.0%) | 1(0.6%) |
| Alanine aminotransferase increased | 19 (11.9%) | 23(14.6%) |
| Aspartate aminotransferase decreased | 0 (0.0%) | 1(0.6%) |
| Aspartate aminotransferase increased | 25 (15.7%) | 23(14.6%) |
| Basophil count increased | 3 (1.9%) | 1(0.6%) |
| Blood albumin decreased | 9 (5.7%) | 13(8.2%) |
| Blood albumin increased | 0 (0.0%) | 1(0.6%) |
| Blood alkaline phosphatase increased | 15 (9.4%) | 15(9.5%) |
| Blood bilirubin decreased | 1 (0.6%) | 0(0.0%) |
| Blood bilirubin increased | 6 (3.8%) | 10(6.3%) |
| Blood creatinine decreased | 0 (0.0%) | 1(0.6%) |
| Blood creatinine increased | 3 (1.9%) | 4(2.5%) |
| Blood glucose decreased | 5 (3.1%) | 6(3.8%) |
| Blood glucose increased | 9 (5.7%) | 9(5.7%) |
| Blood potassium decreased | 2 (1.3%) | 4(2.5%) |
| Blood potassium increased | 14 (8.8%) | 15(9.5%) |
| Blood sodium decreased | 13 (8.2%) | 12(7.6%) |
| Blood urea decreased | 0 (0.0%) | 2(1.3%) |
| Blood urea increased | 1 (0.6%) | 1(0.6%) |
| Electrocardiogram QT prolonged | 3 (1.9%) | 5(3.2%) |
| Eosinophil count decreased | 1 (0.6%) | 0(0.0%) |
| Eosinophil count increased | 33 (20.8%) | 36(22.8%) |
| Globulins increased | 1 (0.6%) | 0(0.0%) |
| Haematocrit decreased | 39 (24.5%) | 37(23.4%) |
| Haemoglobin decreased | 34 (21.4%) | 31(19.6%) |
| Haemoglobin increased | 0(0.0%) | 2(1.3%) |
| Lymphocyte count decreased | 2(1.3%) | 1(0.6%) |
| Lymphocyte count increased | 1(0.6%) | 4(2.5%) |
| Mean cell haemoglobin decreased | 6(3.8%) | 8(5.1%) |
| Mean cell haemoglobin increased | 3(1.9%) | 6(3.8%) |
| Mean cell volume decreased | 3(1.9%) | 9(5.7%) |
| Mean cell volume increased | 3(1.9%) | 1(0.6%) |
| Monocyte count increased | 4(2.5%) | 9(5.7%) |
| Neutrophil count decreased | 7(4.4%) | 11(7.0%) |
| Neutrophil count increased | 2(1.3%) | 1(0.6%) |
| Platelet count decreased | 14(8.8%) | 12(7.6%) |
| Platelet count increased | 1(0.6%) | 2(1.3%) |
| Protein total decreased | 9(5.7%) | 5(3.2%) |
| Protein total increased | 9(5.7%) | 11(7.0%) |
| Red blood cell sedimentation rate increased | 1(0.6%) | 0(0.0%) |
| Reticulocyte count increased | 25(15.7%) | 34(21.5%) |
| White blood cell count decreased (p=0.0018) | 1 (0.6%) | 12 (7.6%) |
| White blood cell count increased | 10(6.3%) | 8 (5.1%) |
| Decreased appetite | 2(1.3%) | 1 (0.6%) |
| Dehydration | 1(0.6%) | 0 (0.0%) |
| Hyperkalaemia | 0(0.0%) | 1 (0.6%) |
| Hypoglycaemia | 0(0.0%) | 2(1.3%) |
| Myalgia (p=0.0448) | 4(2.5%) | 0(0.0%) |
| Headache | 2(1.3%) | 5(3.2%) |
| Nephritis | 1(0.6%) | 0(0.0%) |
| Cough | 1(0.6%) | 2(1.3%) |
| Oropharyngeal pain | 0(0.0%) | 1(0.6%) |
| Hyperhidrosis | 1(0.6%) | 1(0.6%) |
| Pruritus (p=0.0435) | 0(0.0%) | 4(2.5%) |
| Hypotension | 0(0.0%) | 1(0.6%) |
| Note: Significant Chi square p< 0.005 | | |
